# A kinder, gentler dopamine… highlighting dopamine's role in behavioral flexibility

**DOI:** 10.3389/fnins.2014.00004

**Published:** 2014-01-24

**Authors:** Jeff A. Beeler, Roshan Cools, Monica Luciana, Sean B. Ostlund, Giselle Petzinger

**Affiliations:** ^1^Department of Psychology, Queens College and the Graduate Center, CUNYFlushing, NY, USA; ^2^Center for Cognitive Neuroimaging, Donders Institute for Brain, Cognition and BehaviourNijmegen, Netherlands; ^3^Department of Psychology, University of MinnesotaMinneapolis, MN, USA; ^4^Psychiatry and Biobehavioral Science, UCLA Semel Institute for Neuroscience, University of California Los AngelesLos Angeles, CA, USA; ^5^Department of Neurology, University of Southern CaliforniaLos Angeles, CA, USA

**Keywords:** behavioral flexibility, explore-exploit, dopamine, dopamine D2 receptor, decision-making

If dopamine were a politician, it might have an image problem. The organizing metaphor for dopamine function is *reward*. Implicit in this formulation is that a system that drives appetitive pursuit toward needed resources is essential for survival. Dopamine is the go-and-get-it neurotransmitter. In it's association with addiction and compulsive behavior— a “hijacked reward system”— dopamine has become neuroscience's version of Freud's *id*, driving appetitive pursuit without regard to consequences.

However, some evidence suggests that dopamine is not essential for basic reward related behaviors. Even with diminished dopamine transmission, animals still like food, still eat, and can still learn about rewards. Dopamine is not at the root of these functions; rather, it is *modulatory*. That is, dopamine *adjusts* these functions, which begs the question *to what end*? The impulse behind this research topic is that the “root” function of dopamine is not to drive reward behavior, but rather to *adjust* reward oriented behavior in order to achieve *adaptive behavioral flexibility*. That is, dopamine evolved to *adapt* reward pursuit, not blindly drive it like a catecholaminergic id. From this perspective, compulsive pathology arises from the *loss* of dopamine's capacities to adapt appetitive behavior to the environment. Instead of thinking of dopamine as a “reward neurotransmitter,” we might just as well consider it a “flexibility neurotransmitter.” This is not merely semantic but can reframe the questions we ask, how experiments are designed and how data are interpreted. However, conceptual frameworks for thinking about dopamine and behavioral flexibility are not as well developed as those that emphasize dopamine and reward.

The papers in this research topic highlight several themes relevant to viewing dopamine as a neurotransmitter that has evolved to promote flexibility in the service of appetitive pursuit. Fundamental to behavioral flexibility is the ability to strike the right balance between exploiting acquired knowledge and exploring to update one's knowledge, the so-called explore-exploit dilemma, which dopamine may regulate (Beeler et al., 2010; Humphries et al., 2012). Nelson and Killcross (2013), investigating habit formation—the exploit end of this continuum—find that blocking D2 receptors enhances accelerated habit formation associated with amphetamine administration while D1 blockade, in contrast, reverses this effect. These data suggest that diminished D2 signaling may favor exploitation of prior learning and imply, conversely, that D2 promotes behavioral flexibility. This theme is repeated in Barker et al. (2013) study of the role of dopamine in the prefrontal cortex where they observe that *activation* of D2 receptors facilitates flexible behavioral responding and appears necessary for restoring sensitivity to changes in outcome value; that is, necessary for updating value associated with actions and subsequent behavior. Similar to Nelson, Barker found, conversely, that *antagonism* of D1 facilitated flexibility. These studies, together, suggest that D1 may facilitate exploitation of prior learning while D2 may promote behavioral flexibility. Klanker et al. (2013) systematically review the role of dopamine in different aspects of cognitive flexibility. Consistent with Nelson and Barker, D2 signaling again emerges as a key substrate mediating adaptive flexibility. Behavioral flexibility depends upon how information is stored and how it can be accessed and utilized. Eppinger et al. (2013) report a study examining differences between younger and older adults in the utilization of model-based and model-free value representation, reporting that older adults exhibit greater reliance on less flexible model-free strategies.

Of course, the dopamine system is not monolithic. Although we may abstract general themes, as above, other papers in this topic remind us that dopamine contributes to a mosaic of functions. In a review of its role in prefrontal function, Floresco (2013) challenges the widely held notion that the effects of dopamine can be characterized uniformly as an inverted U dose-response function. Instead, he suggests, the interaction between D1 and D2 varies depending upon what aspect of behavioral flexibility is being examined, suggesting that realistic dose-response characterization of dopamine requires a family of functions in which different aspects of behavioral flexibility respond differently to changes in dopamine signaling. While we often focus on dopaminergic modulation of specific targets, van der Schaaf et al. (2013) remind us that these targets do not operate in isolation. As above, the authors found that D2 played a key role in mediating behavioral flexibility (reversal learning), but that it did so in a way that depended on individual differences in anatomical connections between the orbitofrontal cortex and the amygdala. These data highlight that dopamine's effects on behavioral flexibility involve changes in communication between structures.

Baudonnat et al. (2013) take a step back and consider the problem of behavioral flexibility in a more complex, naturalistic context in which part of the challenge faced by the animal is determining which stimuli may or may not be relevant and when to use prior learning, or memory, versus when to pursue new learning. In their review, they focus on elaborating a dopaminergic mechanism—incorporating the different timescales of dopamine action and its modulation of corticostriatal plasticity—by which the animal determines “how surprising” the world is and adjust behavior according to the degree of uncertainty.

Associated primarily with signaling positive, rewarding outcomes, what role does dopamine play in altering behavior in response to deleterious outcomes? Oleson and Cheer (2013) review recent, elegant work in their lab using cyclic voltammetry to characterize changes in dopamine signaling across the course of aversive avoidance conditioning and show that dopamine release in response to cues preceding an aversive stimulus changes as that cue shifts from signaling *fear* to signaling *safety*, once the animal learns to avoid the aversive stimulus, engaging dopamine incentive learning.

Finally, returning to pathology in the dopamine system, Cepeda and Levine (Chen et al., 2013) examine the time course of dopaminergic pathophysiology in Huntington's disease where both hyper- and hypo- dopaminergic pathologies emerge sequentially over time contributing to shifting pathologies in behavioral flexibility. Their detailed review highlights mechanistic questions, consistent with a recurring theme, on the differential contribution of D1 and D2 across this progression. Their analysis reframes critical mechanistic questions and highlights how a more nuanced understanding of dopamine pathophysiology may lead to better therapeutics.

The traditional view of dopamine as the “reward neurotransmitter” has, in recent years, been gradually evolving. Through the work of many, dopamine is increasingly framed in neuroeconomic, decision-making terms (e.g., Gan et al., 2010; Schultz, 2010), shifting emphasis from driving reward pursuit to learning about value associated with stimuli and actions and adapting behavior accordingly. Implicit in this shift is an increasing emphasis on how dopamine mediates adaptive flexibility: rather than being the *id* of neurotransmitters, dopamine emerges more as the brain's chief comptroller in energy allocation (Beeler et al., 2012). We believe much is to be gained from explicitly reframing of dopamine's “root function” not as mediating reward, but as mediating behavioral flexibility in the allocation and pursuit of resources. We hope the contributions in this research topic stimulate thinking about dopamine as the “flexibility neurotransmitter” given its critical role in appetitive motivation.
